# The synergistic interaction of vitamin D deficiency and insomnia on dizziness-related handicap in patients with benign paroxysmal positional vertigo

**DOI:** 10.3389/fneur.2025.1656528

**Published:** 2025-10-15

**Authors:** Bo Tang, Minghua Luo, Dan Wang, Yuqin He, Chuang Zhang, Xiaojun Yu

**Affiliations:** Department of Neurology, The First Hospital of Changsha, Changsha, Hunan, China

**Keywords:** benign paroxysmal positional Vertigo (BPPV), vitamin D deficiency, insomnia, interaction effect, dizziness handicap inventory (DHI)

## Abstract

**Background:**

While insomnia and vitamin D deficiency are known risk factors for BPPV, their interactive effect on the dizziness-related handicap is unknown. Given that both may independently promote pro-inflammatory states, a synergistic interaction is biologically plausible. Therefore, this study aimed to investigate both the independent and interactive effects of insomnia and 25-OH-D levels on the dizziness-related handicap in BPPV patients. We specifically hypothesized that insomnia amplifies the detrimental impact of low vitamin D, aiming to provide an empirical basis for integrated clinical management strategies.

**Methods:**

In this cross-sectional study, 125 patients with BPPV were enrolled. Serum 25-OH-D levels and Dizziness Handicap Inventory (DHI) scores were measured. A multivariable linear regression model, refined by a systematic variable selection procedure, was used to assess the effects of insomnia and 25-OH-D on log-transformed DHI scores after adjusting for potential confounders.

**Results:**

After multivariable adjustment in the final parsimonious model, insomnia, lower 25-OH-D levels, and their interaction term all remained highly significant predictors of higher dizziness-related handicap (all *p* < 0.01). The interaction indicated that the negative association between 25-OH-D and DHI was significantly stronger in patients with insomnia.

**Conclusion:**

Insomnia and vitamin D deficiency are independently associated with greater dizziness-related handicap in BPPV patients, and they demonstrate a significant synergistic interaction. However, due to the study’s design, a definitive causal relationship cannot be established. Assessing and managing both conditions may be crucial for mitigating the handicap imposed by BPPV.

## Introduction

Benign paroxysmal positional vertigo (BPPV) stands as the leading cause of vertigo, accounting for up to 24.1% of cases among patients presenting with dizziness or vertigo (1). The pathophysiology of BPPV is understood to involve the dislodgement of degenerated otoliths into a semicircular canal. While canalithiasis is the most widely accepted mechanism, the pathophysiology is not completely understood, and other theories such as cupulolithiasis and the loss of superior vestibular ganglion cells have also been proposed as contributing factors (2). This displacement pathologically renders the canal sensitive to linear accelerations, such as gravity, during changes in head position. A healthy canal does not respond to this stimulus, but one containing otoconial debris will be inappropriately activated, thereby inducing paroxysmal attacks of positional vertigo (3). The clinical diagnosis of its various subtypes relies on a characteristic history and the observation of specific nystagmus during positional tests, as defined by the consensus criteria of the Bárány Society. With a lifetime incidence reaching 2.4% (4), BPPV significantly impairs the quality of life for affected individuals (5), elevates their risk of falls, and diminishes their walking speed (6). As a result, BPPV imposes a substantial medical burden worldwide (7). Therefore, developing effective strategies to alleviate patient symptoms has become a key objective in clinical research.

The pathogenesis of BPPV is attributed to the dislodgement of otoconia, which are small calcium carbonate crystals, into one of the semicircular canals (most frequently the posterior canal). While this displacement can be precipitated by head trauma, the majority of BPPV cases are idiopathic, and its precise pathophysiology is not yet fully understood (8). Several studies have suggested an association between serum vitamin D levels and the occurrence of BPPV (9–11). However, this link has been challenged by other researchers who argue that the correlation is unproven with existing data, suggesting the coexistence of BPPV and vitamin D deficiency may be merely coincidental (12, 13). Thus, the relationship between vitamin D status and BPPV remains a subject of debate.

Insomnia, a prevalent sleep disturbance defined within the seven major categories of the International Classification of Sleep Disorders (ICSD-3) (14), has recently emerged as a factor potentially associated with BPPV. Some prior research has proposed mechanisms by which insomnia could precipitate BPPV, including the induction of neuroendocrine dysregulation—characterized by elevated cortisol levels and heightened sympathetic nervous system activity (15–17)—as well as the inflammatory activation of vestibular neurons (15, 18). Moreover, insomnia has been linked to an increased risk of BPPV recurrence (19). Despite these preliminary findings suggesting a relationship between sleep disorders and the clinical course of BPPV, the specific impact of insomnia on the dizziness-related handicap of symptoms in these patients has not been thoroughly investigated. Furthermore, the interplay between these factors may be more direct, as emerging evidence suggests vitamin D plays a multifaceted role in sleep regulation itself through neurotransmitter systems, circadian rhythms, and neuroimmune regulation (20).

Patients with BPPV often present with multiple risk factors, whose interplay may be synergistic or antagonistic, extending beyond simple linear effects. However, the interaction between two common factors, insomnia and vitamin D deficiency, and its effect on the dizziness-related handicap of BPPV remains unelucidated. We hypothesize that a significant interaction exists between insomnia and vitamin D deficiency, which collaboratively exacerbates the dizziness-related handicap of BPPV. To test this hypothesis, we utilized multivariable linear regression modeling, refined by a systematic variable selection procedure, to analyze the combined effect of these two factors via an interaction term. The aim of this study was to provide an empirical basis for precise clinical interventions in BPPV management.

## Methods

### Participants

In this study, a total of 125 patients with BPPV were recruited from the Department of Neurology at The First Hospital of Changsha between January and June 2025. The diagnosis of BPPV was confirmed for each patient by a certified neurologist based on the diagnostic criteria of the Bárány Society (21). The exclusion criteria included: (1) a history of other peripheral vestibular diseases, such as Meniere’s disease; (2) accompanied by new-onset or fluctuating hearing impairment(a criterion intended to exclude alternative diagnoses, thereby permitting the inclusion of patients with stable, pre-existing hearing loss such as presbycusis); (3) head trauma within the preceding month; (4) systemic musculoskeletal disorders; (5) severe organic diseases; and (6) any central causes of vertigo, including vestibular migraine, intracranial space-occupying lesions, or cerebrovascular malformations. This study was conducted in accordance with the principles of the Declaration of Helsinki. The study protocol was reviewed and approved by the Ethics Committee of The First Hospital of Changsha.

## Data collection and variables

### Primary outcome: the dizziness-related handicap of BPPV

The Dizziness Handicap Inventory (DHI) is a commonly employed, 25-item self-report questionnaire designed to quantify the impact of dizziness on a patient’s life. The DHI was administered to all patients at the time of diagnosis, before any therapeutic repositioning maneuvers were performed, to establish a baseline measure of the dizziness-related handicap. It assesses the perceived handicap across three key dimensions: physical, functional, and emotional (22, 23). The total score is calculated on a scale from 0 (no handicap) to 100 (severe disability), with higher values corresponding to greater functional limitations and a more significant negative impact on quality of life. Recognized as a benchmark tool for measuring dizziness-related impairment, the DHI has well-established psychometric properties and has been validated in numerous clinical populations (24), including those with BPPV (25).

### Primary predictors

Serum 25-OH-D levels were measured for all participants. For descriptive purposes, a level below 20 ng/mL was classified as vitamin D deficiency, a widely used threshold for skeletal health (26). However, we acknowledge the ongoing discussion regarding optimal levels for extra-skeletal benefits, with some guidelines suggesting a higher threshold of <30 ng/mL for insufficiency (27). To avoid reliance on a single cut-point, our primary multivariable regression analysis utilizes 25-OH-D level as a continuous variable.

The presence of insomnia was assessed using criteria from the World Mental Health Survey Initiative Version of the WHO Composite International Diagnostic Interview (WMH-CIDI) (28). All participants were questioned about their sleep patterns over the preceding 12 months, specifically concerning any period lasting 2 weeks or longer. The assessment focused on three core symptoms: (1) difficulty initiating sleep (defined as taking two or more hours to fall asleep almost nightly); (2) difficulty maintaining sleep (waking up almost nightly and being unable to return to sleep for an hour or more); and (3) early morning awakening (waking up at least 2 h earlier than planned almost every morning). Participants who responded affirmatively to at least one of these three items were categorized into the insomnia group, while those who answered negatively to all three were placed in the no-insomnia group.

### Covariates

Other participant characteristics were collected through a standardized questionnaire, including age, gender, body mass index (BMI), smoking status, and alcohol consumption. The presence of comorbidities such as hypertension and diabetes was ascertained from medical records.

## Statistical analysis

All statistical analyses were performed using R software (version 4.5.0). A two-sided *p*-value less than 0.05 was considered statistically significant.

The normality of distribution for continuous variables was assessed using the Shapiro–Wilk test. Baseline characteristics were compared between the insomnia and no-insomnia groups. The independent *t*-test was used for normally distributed continuous variables (reported as mean ± SD), while the Mann–Whitney U test was used for non-normally distributed variables (reported as median [IQR]). Categorical variables (reported as frequency and percentage) were compared using Pearson’s chi-squared test or Fisher’s exact test, as appropriate.

To identify factors associated with dizziness-related handicap, a multivariable linear regression analysis was performed. The Dizziness Handicap Inventory (DHI) score, the primary outcome, was found to be right-skewed and was therefore log-transformed [log(DHI + 1)] to better meet the assumptions of normality and homoscedasticity of residuals.

A systematic variable selection procedure was employed to build a parsimonious model and mitigate the risk of overfitting. All clinically relevant covariates (Age, BMI, Gender, Smoke, Alcohol drinking, Hypertension, Diabetes), along with the primary predictors (Insomnia and 25-OH-D) and their interaction term, were entered into an initial model. A backward elimination strategy was then applied, where variables with the highest *p*-value > 0.10 were iteratively removed. Collinearity was assessed for the final model using the Variance Inflation Factor (VIF). Model fitness was evaluated using the Adjusted R-squared value.

## Results

A total of 125 participants were included in this study, comprising 67 (53.6%) in the insomnia group and 58 (46.4%) in the no-insomnia group. The detailed baseline demographic and clinical characteristics of all participants are presented in Table 1.

**Table 1:** Baseline characteristics of the study participants stratified by insomnia status.

Characteristic	Overall	No	Yes	*p*-value
Gender (Female), n (%)	34 (27.2%)	20 (34.5%)	14 (20.9%)	0.091
Age (years), mean (SD)	57.5 (13.9)	57.0 (14.2)	58.1 (13.6)	0.598
BMI (kg/m^2^), mean (SD)	23.6 (3.4)	23.3 (3.0)	23.9 (3.7)	0.422
Smoke, n (%)	18 (14.4%)	4 (6.9%)	14 (20.9%)	0.033
Alcohol drinking, n (%)	6 (4.8%)	0 (0%)	6 (9.0%)	0.031
Hypertension, n (%)	39 (31.2%)	6 (10.3%)	33 (49.3%)	<0.001
Diabetes, n (%)	14 (11.2%)	2 (3.4%)	12 (17.9%)	0.010
25-OH-D (ng/mL), median [IQR]	16.8 [12.2–22.1]	19.1 [14.5–22.4]	14.4 [11.6–21.0]	0.024
DHI, median [IQR]	34.0 [22.0–56.0]	22.0 [16.5–24.0]	54.0 [48.0–60.0]	<0.001
Vit D Insufficiency (<30 ng/mL), n (%)	105 (84.0%)	44 (75.9%)	61 (91.0%)	0.027

A comparison of baseline data revealed no statistically significant differences between the two groups in terms of age, BMI, or gender distribution (all *p* > 0.05). However, significant differences were observed in lifestyle factors, comorbidities, and serum 25-OH-D levels. Specifically, the insomnia group had a significantly higher proportion of smokers (20.9% vs. 6.9%, *p* = 0.033) and alcohol drinkers (9.0% vs. 0%, *p* = 0.031) compared to the no-insomnia group.

The prevalence of both hypertension (49.3% vs. 10.3%, *p* < 0.001) and diabetes (17.9% vs. 3.4%, *p* = 0.01) was significantly greater in the insomnia group. Concurrently, patients with insomnia exhibited significantly lower mean serum 25-OH-D levels than those without insomnia (14.4 ng/mL [IQR: 11.6–21.0] vs. 19.1 ng/mL [IQR: 14.5–22.4], *p* = 0.024). Furthermore, when using a higher threshold of <30 ng/mL to define vitamin D insufficiency, the prevalence of insufficiency was significantly greater in the insomnia group compared to the no-insomnia group (91.0% vs. 75.9%, *p* = 0.027).

**Figure 1:**
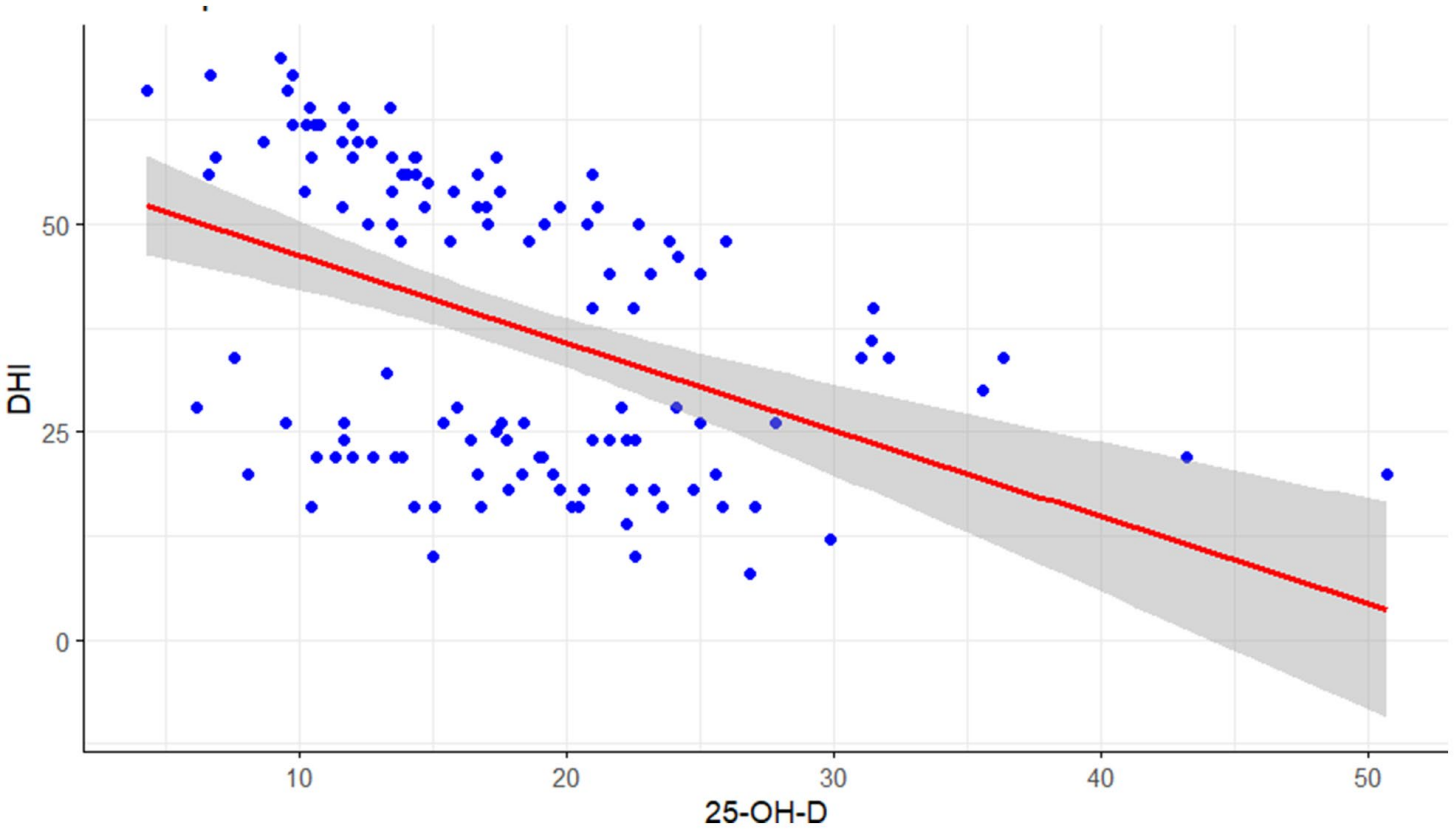
Scatter plot of DHI scores against vitamin D levels.

The direct relationship between 25-OH-D levels and DHI scores was then examined. As illustrated in the scatterplot in Figure 1, a Spearman rank correlation analysis revealed a statistically significant, moderate negative correlation between the two variables (*r* = −0.44, *p* < 0.001), indicating that higher vitamin D levels are associated with lower dizziness-related handicap.

**Figure 2:**
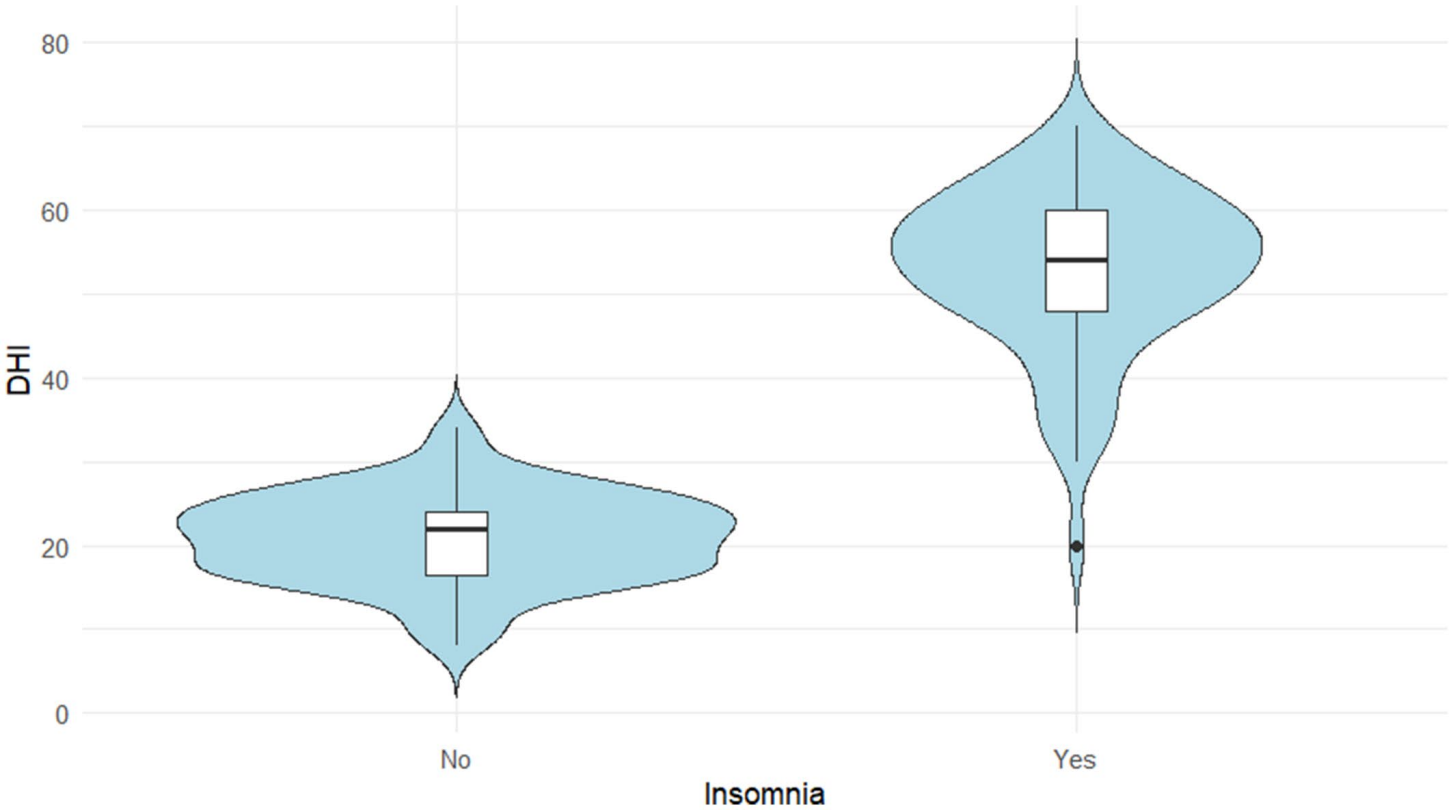
Distribution of DHI scores according to insomnia status.

To assess the impact of insomnia, DHI scores were compared between patients with and without insomnia. A Mann–Whitney U test revealed that the DHI scores were significantly higher in the insomnia group compared to the non-insomnia group (*p* < 0.05). The violin plot in Figure 2 visually illustrates this difference in score distribution between the two groups.

A multivariable linear regression model was built to identify predictors of the log-transformed DHI score. After applying a backward elimination procedure, the final parsimonious model retained age, gender, 25-OH-D level, insomnia status, and the interaction term between insomnia and 25-OH-D. The results of this final model are presented in Table 2. Collinearity was not detected among the variables in the final model (all VIF < 2). The model explained a significant portion of the variance in log-DHI scores (Adjusted R^2^ = 0.73, F-statistic *p* < 0.001).

**Table 2:** Multivariable linear regression analysis of factors associated with the log-transformed DHI score.

Characteristic	*β*	95% CI	*p*-value
Age (per year)	−0.01	−0.02, 0.00	0.052
Gender (Male vs. Female)	0.49	0.26, 0.72	<0.001
Insomnia (Yes vs. No)	2.15	1.83, 2.47	<0.001
25-OH-D (per ng/mL)	−0.02	−0.03, −0.00	0.015
Insomnia (Yes vs. No) * 25-OH-D (per ng/mL)	−0.05	−0.07, −0.03	<0.001

Critically, even in this more robust model, both insomnia (*β* = 2.15, *p* < 0.001) and 25-OH-D level (*β* = −0.02, *p* = 0.015) remained significant independent predictors. Most importantly, the synergistic interaction between insomnia and 25-OH-D also remained highly significant (*β* = −0.05, *p* < 0.001), confirming the primary finding of this study.

## Discussion

BPPV is one of the most common peripheral vestibular disorders (7). Although its diagnosis and treatment via physical repositioning maneuvers are well-established (3), a substantial proportion of patients continue to experience recurrent or residual symptoms (19, 29), which significantly impairs their quality of life. It has long been recognized in clinical practice that the pathogenesis and recovery process of BPPV is multifactorial, extending beyond a single pathological mechanism (7). Against this background, the present study incorporated two highly prevalent factors in BPPV patients, vitamin D deficiency and insomnia, into a unified analytical framework. We not only confirmed their respective independent effects but, more importantly, revealed for the first time a significant statistical interaction between them, which jointly exacerbates the dizziness-related handicap in patients with BPPV. This finding offers a novel perspective for understanding the pathophysiology of BPPV and holds important implications for the optimization of clinical management strategies.

A finding from our baseline analysis is that patients with insomnia presented with a significantly less favorable clinical profile, characterized by a higher prevalence of smoking, alcohol consumption, hypertension, and diabetes, alongside lower serum 25-OH-D levels. This clustering of risk factors is consistent with extensive evidence from epidemiological studies, which has firmly established a link between chronic sleep disturbances and poor cardiometabolic and endocrine health. The underlying pathways are likely multifactorial, involving sympathetic nervous system overactivity, hypothalamic–pituitary–adrenal (HPA) axis dysregulation, and pro-inflammatory states common in insomnia.

From a methodological standpoint, the significant imbalance of these variables at baseline underscores their role as powerful potential confounders. Had these differences not been accounted for, any observed association between insomnia and dizziness could have been biased. Therefore, our analytical strategy—which involved including all these potential confounders in an initial model before applying a systematic variable selection procedure—was an essential step. This process ensured that we isolated the independent effects of insomnia and vitamin D in a robust yet parsimonious final model, thereby strengthening the validity and reliability of our main findings.

Our multivariate linear regression analysis revealed a significant inverse correlation between serum 25-OH-D levels and log-transformed DHI scores (*β* = −0.02, *p* = 0.015), even after adjusting for potential confounders including smoking, alcohol consumption, hypertension, and diabetes. This indicates that lower vitamin D levels are associated with greater dizziness-related handicap. This finding is consistent with a substantial body of previous research, which has established a crucial role for vitamin D in maintaining inner ear calcium homeostasis and normal otoconial metabolism (30). The pathophysiological mechanism is understood to involve the active form of vitamin D, 1,25-(OH)₂D₃, which not only regulates systemic calcium absorption but is also thought to act directly on the inner ear, influencing the formation and decomposition of the otoconial membrane (31). Consequently, vitamin D deficiency can lead to otoconial decalcification and structural abnormalities, increasing their fragility and susceptibility to dislodgement, thereby elevating the risk of BPPV. Furthermore, low levels of vitamin D and calcium may lead to demineralization of the otic capsule and cause degenerative changes in the spiral ligament, stria vascularis, and cochlear hair cells (32).

Our research extends beyond the established role of vitamin D deficiency in the pathogenesis of BPPV to investigate its association with dizziness-related handicap. Our findings suggest that vitamin D’s role extends beyond that of a mere initiating factor; a state of chronic deficiency may exacerbate symptoms through multiple interconnected pathways. Firstly, persistent dysregulation of calcium metabolism can not only lead to the dislodgement of otoconia (10) but also impair their degeneration-regeneration cycle, hindering the effective clearance or dissolution of canalith debris (33). This, in turn, may prolong the duration and frequency of vertiginous episodes. Secondly, vitamin D is recognized as an important neurosteroid with widespread neuroprotective and anti-inflammatory effects (34). A deficiency in vitamin D may therefore impair the central nervous system’s plasticity and capacity for vestibular compensation, making it difficult for patients to adapt to changes in vestibular function after successful repositioning maneuvers and leading to the persistence of residual symptoms such as dizziness and instability (29). Furthermore, the well-established link between vitamin D deficiency and emotional disorders such as depression and anxiety introduces another dimension (35), as these psychological factors are known to act as potent amplifiers of subjective dizziness (36, 37).

In addition to vitamin D deficiency, insomnia was confirmed as another powerful independent predictor of increased dizziness-related handicap (*β* = 2.15, *p* < 0.001), a finding that aligns with existing literature. A primary proposed mechanism is the disruption of central vestibular compensation. The neuroplastic processes within the cerebellum and cerebral cortex required for restoring balance after a vestibular insult rely heavily on healthy sleep cycles, particularly REM and slow-wave sleep, for consolidation. Insomnia hinders this critical period of neural repair, thereby delaying functional recovery and leading to the persistence of symptoms (38, 39). Also, insomnia is recognized as a state of hyperarousal that leads to dysregulation of the HPA axis and over-activation of the sympathetic nervous system, resulting in elevated levels of circulating stress hormones, such as cortisol (40). These neuroendocrine alterations are thought to directly influence the excitability of the vestibular nuclei, lowering the system’s stability threshold. Consequently, patients may exhibit an exaggerated vertiginous response to minor positional changes or otherwise benign vestibular stimuli (41). Furthermore, insomnia is closely linked to a pro-inflammatory state. A substantial body of evidence indicates that sleep disturbances promote systemic low-grade inflammation, characterized by elevated levels of pro-inflammatory cytokines such as tumor necrosis factor-*α* (TNF-α) and interleukin-6 (IL-6) (42). These inflammatory mediators may not only cause direct damage to inner ear hair cells and vestibular neurons (43) but can also affect central nervous system function via the blood–brain barrier (44), collectively exacerbating vestibular dysfunction.

A crucial consideration in interpreting these findings is the direction of causality. While we have discussed the mechanisms through which insomnia may exacerbate the dizziness-related handicap of BPPV, our cross-sectional design does not allow us to establish a definitive causal link. This causal ambiguity also extends to the relationship between vitamin D and insomnia itself; while low vitamin D may contribute to poor sleep through the pathways discussed, it is also plausible that the lifestyle and physiological changes associated with chronic insomnia could lead to reduced sun exposure and altered vitamin D metabolism. It is entirely plausible, as has been suggested, that the relationship is bidirectional. The experience of BPPV itself—particularly the occurrence of vertiginous episodes when turning in bed at night—and the anticipatory anxiety about triggering an attack can significantly disrupt sleep architecture and lead to the development or worsening of insomnia. Therefore, a “vicious cycle” may exist, in which insomnia impairs vestibular compensation, leading to more severe symptoms, which in turn fuels further sleep disturbances. Future prospective, longitudinal studies are necessary to untangle this complex, bidirectional relationship and to determine the temporal sequence of these events.

The core finding of this study is the significant statistical interaction between vitamin D deficiency and insomnia in influencing dizziness-related handicap (*β* for interaction = −0.05, *p* < 0.001). This indicates that the combined negative impact of these two factors is not merely additive but rather synergistic. Specifically, our final model demonstrates that the negative association between 25-OH-D levels and the log-transformed DHI score was substantially amplified in patients with insomnia. While the effect of each 1 ng/mL increase in 25-OH-D was modest in the no-insomnia group (main effect *β* = −0.02), this detrimental effect was more than tripled in the presence of insomnia (combined effect *β* = −0.07). This quantitatively demonstrates that insomnia acts as a potent amplifier of the adverse impact of low vitamin D levels on dizziness-related handicap.

The biological underpinnings of this synergistic interaction are likely complex, and we postulate that dysregulation across the neuro-endocrine-immune network may serve as a crucial link. As previously discussed, both vitamin D deficiency and insomnia can independently promote a pro-inflammatory state characterized by elevated cytokines (18, 45, 46). It is plausible that when these conditions coexist, they converge on shared inflammatory signaling pathways, creating a potential ‘inflammatory cascade’ that results in more severe vestibular system damage. Vitamin D is a known potent modulator of the immune system (45, 47), and its deficiency may impair the body’s ability to suppress the pro-inflammatory state induced by insomnia, leading to an uncontrolled inflammatory response (45, 48).

Furthermore, a second potential pathway for this synergy may involve the central nervous system. Vitamin D is involved in regulating the synthesis of key neurotransmitters, including serotonin (49) and dopamine (50), which are critical for modulating mood and sleep and also participate in central vestibular processing (51, 52). A hypothesis requiring further investigation is that a deficiency in vitamin D could lead to neurotransmitter imbalances that diminish the central nervous system’s capacity to compensate for the hyperarousal and HPA-axis dysregulation induced by insomnia (53). Therefore, it is conceivable that vitamin D deficiency and insomnia create a vicious cycle through converging inflammatory and neurochemical pathways, collectively impairing the robustness and restorative capacity of the vestibular system and ultimately leading to more severe clinical symptoms. These proposed mechanisms are speculative and await direct empirical validation in future studies.

The primary strength of this study lies in its novelty. To our knowledge, this is the first study to investigate the combined effect of vitamin D deficiency and insomnia on dizziness-related handicap using a statistical interaction model. Furthermore, the reliability of our conclusions is enhanced by our rigorous statistical approach. We employed a range of analytical methods—from univariate comparisons and correlation analysis to a sophisticated multivariable modeling strategy that involved log-transformation of the outcome and a systematic variable selection procedure, to validate our hypothesis from multiple perspectives.

An additional noteworthy finding from our final multivariable model was that male gender was significantly associated with a greater dizziness-related handicap. This is an interesting result, as some epidemiological studies on dizziness report a higher prevalence and symptomatic burden in females, potentially due to hormonal influences or differences in healthcare-seeking behavior (54). However, other studies have found no significant gender differences in outcomes after treatment for BPPV. Our finding might suggest that, within this specific BPPV cohort, men who suffer from both insomnia and low vitamin D may experience a uniquely impactful handicap, although the underlying mechanism is unclear and could be related to unmeasured psychosocial factors. This observed gender difference warrants further investigation in future studies (55).

This study has several limitations that should be acknowledged. First and foremost, the cross-sectional design precludes any definitive establishment of causality. The observed associations do not prove directionality, and bidirectional relationships—particularly among BPPV-related handicap, insomnia, and vitamin D status—are plausible and require future longitudinal studies to untangle. Second, there are limitations related to the measurement of variables and uncollected data. The most significant of these is the lack of a formal assessment for mental health status. The DHI score is known to be associated not only with the direct physical impact of vestibular disease but also with broader mental health and quality-of-life measures (23). More specifically, it has been suggested that while lower DHI scores often relate to structural vestibular disorders alone, the very high scores (>60) seen in a large portion of our insomnia group are likely to involve co-existing functional or psychiatric disorders (56). Given the high comorbidity between insomnia, anxiety, and depression, the absence of this data means that an unmeasured psychiatric condition remains a key potential confounder for our findings. This is compounded by our assessment of insomnia, which relied on a self-report screening instrument (WMH-CIDI) rather than objective measures like polysomnography. This method is susceptible to recall bias and does not capture the full spectrum of sleep disturbances, such as sleep architecture, nor does its dichotomous nature permit a dose–response analysis. Furthermore, we did not collect data on other potentially relevant factors such as dyslipidemia, detailed clinical characteristics of the BPPV presentation (e.g., laterality, detailed symptom evolution, and previous treatment history), or a quantitative assessment of tobacco consumption (e.g., pack-years). Finally, the generalizability of our findings may be limited by the single-center design and moderate sample size. We also acknowledge that the definition of an optimal serum 25-hydroxyvitamin D level remains a subject of scientific debate. While our descriptive analysis used the conventional <20 ng/mL threshold for deficiency, our primary findings regarding the synergistic interaction are derived from a multivariable model that treats vitamin D levels as a continuous variable. This approach has the significant advantage of being independent of any single, disputed cut-point, thereby strengthening the robustness of our main conclusion. The lack of biomarker data also prevented the direct validation of our proposed biological mechanisms, and alternative statistical modeling strategies could be explored in larger future studies.

Despite its limitations, our study has significant clinical implications. We recommend that clinicians routinely screen BPPV patients, particularly those with recurrent or persistent symptoms, for both vitamin D deficiency and insomnia. Patients with both conditions should be considered a high-risk group for more severe dizziness. This points to a potential combination therapy strategy: adding vitamin D supplementation and effective sleep management to standard repositioning maneuvers. Future research should confirm our findings in larger, prospective studies and use randomized controlled trials (RCTs) to test if this combined approach is superior to single treatments. Further studies are also needed to explore the underlying biological mechanisms, such as the proposed neuro-endocrine-immune network.

## Conclusion

Building upon the established relationship between vitamin D and sleep physiology, this study provides new clinical evidence identifying both vitamin D deficiency and insomnia as independent predictors of dizziness-related handicap in BPPV. Critically, our findings also reveal a significant synergistic interaction between them. However, it is important to acknowledge that due to the cross-sectional design of our study, a definitive direction of causality cannot be established. Nevertheless, these findings underscore the necessity of a multidimensional clinical approach to the assessment and management of patients with BPPV.

## Data Availability

The raw data supporting the conclusions of this article will be made available by the authors, without undue reservation.
